# Integration analysis of transcriptome and proteome profiles brings new insights of somatic embryogenesis of two eucalyptus species

**DOI:** 10.1186/s12870-024-05271-6

**Published:** 2024-06-15

**Authors:** Shengkan Chen, Dongqiang Guo, Ziyu Deng, Qinglan Tang, Changrong Li, Yufei Xiao, Lianxiang Zhong, Bowen Chen

**Affiliations:** Guangxi Key Laboratory of Superior Timber Trees Resource Cultivation, Guangxi Forestry Research Institute, 23 Yongwu Road, Nanning, 530002 Guangxi China

**Keywords:** Somatic embryogenesis, Callus, Eucalyptus, Transcription factor, Embryo callus

## Abstract

**Background:**

Somatic embryogenesis (SE) is recognized as a promising technology for plant vegetative propagation. Although previous studies have identified some key regulators involved in the SE process in plant, our knowledge about the molecular changes in the SE process and key regulators associated with high embryogenic potential is still poor, especially in the important fiber and energy source tree – eucalyptus.

**Results:**

In this study, we analyzed the transcriptome and proteome profiles of *E. camaldulensis* (with high embryogenic potential) and *E. grandis x urophylla* (with low embryogenic potential) in SE process: callus induction and development. A total of 12,121 differentially expressed genes (DEGs) and 3,922 differentially expressed proteins (DEPs) were identified in the SE of the two eucalyptus species. Integration analysis identified 1,353 (131 to 546) DEGs/DEPs shared by the two eucalyptus species in the SE process, including 142, 13 and 186 DEGs/DEPs commonly upregulated in the callus induction, maturation and development, respectively. Further, we found that the trihelix transcription factor ASR3 isoform X2 was commonly upregulated in the callus induction of the two eucalyptus species. The SOX30 and WRKY40 TFs were specifically upregulated in the callus induction of *E. camaldulensis*. Three TFs (bHLH62, bHLH35 isoform X2, RAP2-1) were specifically downregulated in the callus induction of *E. grandis x urophylla*. WGCNA identified 125 and 26 genes/proteins with high correlation (Pearson correlation > 0.8 or < -0.8) with ASR3 TF in the SE of *E. camaldulensis* and *E. grandis x urophylla*, respectively. The potential target gene expression patterns of ASR3 TF were then validated using qRT-PCR in the material.

**Conclusions:**

This is the first time to integrate multiple omics technologies to study the SE of eucalyptus. The findings will enhance our understanding of molecular regulation mechanisms of SE in eucalyptus. The output will also benefit the eucalyptus breeding program.

**Supplementary Information:**

The online version contains supplementary material available at 10.1186/s12870-024-05271-6.

## Background

Eucalyptus is a highly diverse genus with more than 660 species belonging to the myrtle family (*Myrtaceae*). It is native to the Tasmania of Australia and surrounding islands, and now widely planted across the world for its increasing importance for timber and pulp [1]. In addition to the natural regeneration, which relies on seeds and is always a slow process due to the length of the juvenile phase pulp [1], eucalyptus can propagate vegetatively, including somatic embryogenesis (SE) and organogenesis in plant tissue culture [2]. The somatic embryos have the bipolar structure, of which the apical and basal meristems can form shoot and root, respectively, and SE is a process in which an embryo (usually derived from a single somatic cell) can regenerate as a whole plant later [3]. Although SE has been applied with the eucalyptus breeding program for years and many studies have been demonstrated [1, 4–10]. The molecular mechanisms and key regulators involved in eucalyptus SE are not well known.

The omics landscape is now extended to include genomics, transcriptomics, epigenomics, proteomics, metabolomics, and single-cell genomics/transcriptomics [11]. Many studies have used the omics technologies to identify key pathways and important regulators in plant SE. For example, Li and colleagues used transcriptome sequencing and identified key photosynthesis and photosynthesis antenna protein pathways and the *LHCB1* gene associated with SE in tea plant [12]. Wang *et al.* identified auxin signaling factors (e.g., AUX-like1, GH3.1, SAUR32-like, IAA9-like, IAA14-like, IAA27-like, IAA28-like and ARF5-like) significantly enriched during the SE of rubber tree (*Hevea brasiliensis*) [13]. They also identified transcription factors (TFs), including WRKY40, WRKY70, MYBS3-like, MYB1R1-like, AIL6 and bHLH93-like, specifically enriched in the late SE stage. Zhang *et al.* identified the upregulation of auxin synthesis-related genes (e.g, *GH3.6* and *PCO2*) and the downregulation of cytokinin dehydrogenase (*CKX6 and CYP450*) genes in the SE of in *Paeonia ostii* 'Fengdan' using transcriptome sequencing technology [14]. Hesami and colleagues used transcriptome sequencing and identified 1,850 genes downregulated and 1,873 genes upregulated in embryogenic callus compared to non-embryogenic callus of cannabis [15], including 42 different TF classes (e.g., *MYB, C2H2, ERF, AP2, bHLH, bZIP, B3, WRKY and GRAS*). Hou *et al.* used LC-MS/MS (liquid chromatography with tandem mass spectrometry) and identified 6,269 differentially expressed proteins (DEPs) in three stages (the primary embryogenic callus, the single embryo, and the cotyledon embryo) of SE in *Larix olgensis*, including 18 TF families were identified, including 18 TFs from the AP2, NAM and WOX families [16]. Zhang et al. identified 1,178 DEPs in the early SE of longan (*Dimocarpus longan*) and found that sodium butyrate (a deacetylase inhibitor) can reduce the proliferation and delay the differentiation of embryogenic callus by regulating the homeostasis of reactive oxygen species (ROS) andindole-3-acetic acid (IAA) [17]. Furthermore, many other TFs have also been identified for the SE in plant, such as LEA (late embryogenesis abundant), SERK (somatic embryogenesis receptor-like kinase), LEC (leafy cotyledon) and WUS (WUSCHEL) [13, 18].

Our lab has been working on the SE of eucalyptus for years. Previously, we reported key miRNAs [9], genes [8, 18], and proteins [10] involved in the SE of two eucalyptus species – *E. camaldulensis* (with high embryogenic potential) and *E. grandis x urophylla* (with low embryogenic potential). However, a systematic analysis is required to integrate these data and to identify key regulators such as TFs for eucalyptus SE. In the present study, we generate new proteomics data of the callus development of the two eucalyptus species and performed deep analysis. Some key TFs that are related to the high embryogenic potential and involved in the SE process of the two eucalyptus species are identified. This is the first time to integrate multiple omics technologies to investigate the early and late SE in eucalyptus. Our findings will bring new insights into the molecular mechanisms and gene regulations for plant SE studies, especially eucalyptus. Further, the output will benefit the eucalyptus breeding program.

## Methods

### Plant material and callus induction

**Fig. 1:**
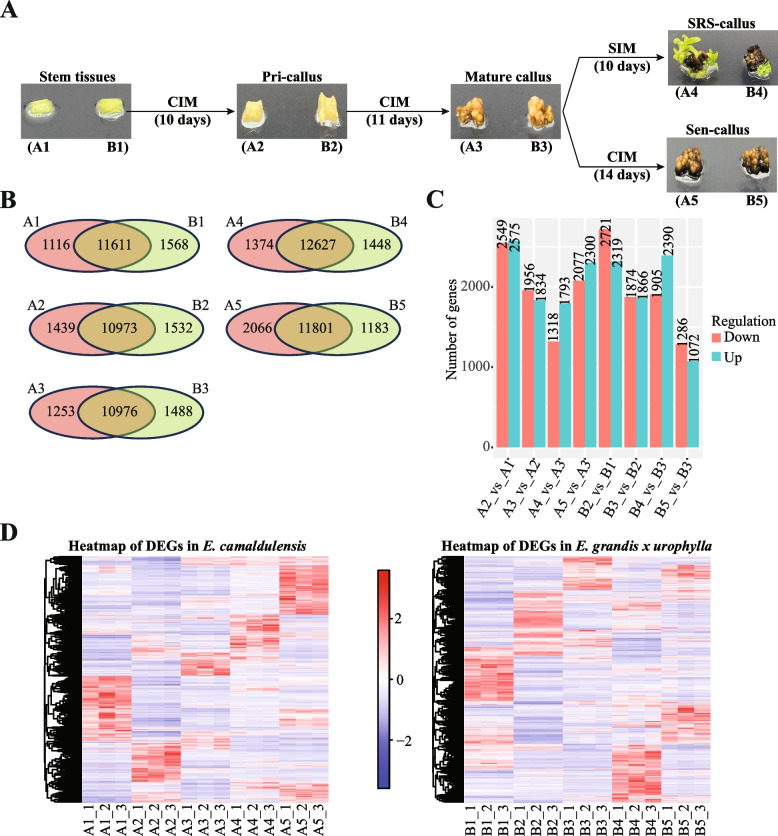
Transcriptome sequencing of two eucalyptus species during SE. **A** Study design of the callus induction and development of the two eucalyptus species. Initially, the stem tissues **A**1, **B**1 were incubated on CIM to differentiate to pri-callus **A**2, **B**2. Then, the pri-callus were incubated on CIM to mat-callus **A**3, **B**3. Mat-calluses were transferred onto SIM for 10 days incubation to expand the buds A4, B4, taken as the SRS-callus. While the mat-calluses incubated on the CIM for 14 days would lose the regeneration ability, which were called ses-callus **A**5, **B**5. Pri-callus: primary callus; mat-callus: mature callus; SRS-callus: shoot regeneration stage callus; sen-callus: senescence callus. **B** Venn diagrams of genes identified in the two eucalyptus species at each stage of the SE process. **C** Number of DEGs identified in the SE of two eucalyptus species. **D** Heat maps of DEGs identified in the SE of two eucalyptus species. A and B represent the tissues of *E. camaldulensis* and *E. grandis x urophylla*

The original seeds of *E. camaldulensis* and *E. grandis* x *urophylla* were obtained in 1984 from the wild without any restrictions. The seeds were then planted in the experimental fields of Guangxi Forestry Research Institute and confirmed by the senior botanist Prof. Dongyun Xiang. To study the molecular changes during the somatic embryogenesis, we used the branches of *E. camaldulensis* (A) and *E. grandis* x *urophylla* (B) as explants to induce subcultured seedlings, which were further used as the initial experimental material to get stem tissues, primary callus (pri-callus), mature callus (mat-callus), shoot regeneration stage callus (SRS-callus) and senescence callus (sen-callus) (Fig. 1A).

Stem tissues: the stem segments (without axillary buds) of subsequent seedlings were cultured with the subculture medium (MS medium supplemented with 20mg/L Ca(NO_3_)_2_, 0.6 mg/L 6-BA, 0.2 mg/L NAA and 0.1 mg/L IAA) and cut into ~0.5 cm pieces (*n*=300). Such stem tissues were marked as A1 and B1 for the two eucalyptus species.

Pri-callus: the stem tissues (A1 and B2) were cultured with the callus induction medium (CIM, MS medium supplemented with 20mg/L Ca(NO3)_2_, 1 mg/L KT and 0.5 mg/L 2,4-D) in dark at 28±2℃ for another 10 days. Successful callus induction was recognized with the tissue enlargement in a dumbbell to rod shape, light yellow to light green color, and moist surface. The pri-callus induced from A1 and B1 were marked as A2 and B2.

Mat-callus: the pri-callus were then cultured with the CIM in dark at 28±2℃ for another 11 days until the appearance of pale yellow to brownish yellow bead like protrusions on the surface of callus. Such callus tissues were recognized as mat-callus and marked as A3 and B3.

SRS-callus: the mat-callus were transferred on to the shooting-inducing medium (SIM, MS medium supplemented with 20 mg/L Ca (NO_3_)_2_, 2.0 mg/L 6-BA and 0.2 mg/L NAA) and cultured under light at 28±2℃ for 10 days to produce light green, leaf spreading, and non-vitrified buds. Such callus tissues were marked as A4 and B4.

Sen-callus: the mat-callus were continually cultured with the CIM in dark at 28±2℃ for 14 days to sen-callus. Sen-callus are brown to dark brown in color but not yet browning to death. Such callus were marked as A5 and B5.

The samples of each stage used for this study were prepared in batches according to the cultivation time during the experiment, so that all samples can be collected at the same time. Then, they were frozen in liquid nitrogen for subsequent testing.

## Transcriptome sequencing and data analysis

We first extracted the total RNA from the plant tissues (A1~A5, B1~B5) using the TRIzol reagent, as previously described [8, 19]. Then, the total RNA was quantified and quality controlled by the Agilent 2100 Bioanalyzer. We used equal amount (1 μg) of total RNA of each sample for the cDNA library preparation and transcriptome sequencing (paired-end 150, PE150) on the BGISEQ-500 platform, as described [8]. The raw reads were cleaned by SOAPnuke to remove sequencing adapters, low quality reads and contamination reads [20]. Then, Hisat2 was used to align the clean reads to the reference eucalyptus genome (v2.0, https://plantgenie.org). We used Stringtie [21] and FeatureCount [22] to profile the gene expression and to count the reads mapped to each gene, respectively. We used the TPM (transcripts per million reads mapped) method to normalized the gene expression across samples and lowly expressed genes were filtered (< 5 TPM). Genes with differential expression during the callus induction and development were identified using the criterial: coefficient of variation (CV) < 0.5, log2 fold change (log2FC) > 1 or < -1, *p*-value < 0.05 and false discovery rate (FDR) < 0.05.

## DIA proteomics analysis of the samples

The protein samples (100 mg) of all samples (A1~A5, B1~B5) were extracted and analyzed by the DIA proteomics strategy, as previously described [10]. The data dependent acquisition (DDA) MS data were analyzed using the MaxQuant (v1.5.3.30) and the NCBI non-redundant eucalyptus protein sequences (44,589 sequences) were used as the reference [23]. The search parameters were same as our previous study [10]. Differentially expressed proteins were identified using the MSstats (log2FC > 1 or < -1, adjusted *p*-value < 0.05) [24].

## Enrichment analysis of KEGG pathway and gene ontology

We first annotated the eucalyptus genes/proteins by mapping them to the Gene Oncology (GO) and Kyoto Encyclopedia of Genes and Genomes (KEGG) pathway databases. Then, enriched GO and KEGG pathway items by the DEGs/DEPs were identified using the *p*-value (< 0.05), calculated by Fisher’s exact test, and q-value (< 0.05), calculated by the ‘qvalue’ package, with the R software, as previously described [25].

## Weighted correlation network analysis

We used the R package “WGCNA” to identify co-expressed genes during the callus development of the two eucalyptus species [26], according to the manufacturer’s protocol.

## qRT-PCR

We validated the expression levels of Eucgr.B03553 and six of its correlated genes (Eucgr.A01153, Eucgr.D00321, Eucgr.D00322, Eucgr.E01768, Eucgr.I00879 and Eucgr.K02925) using quantitative real-time PCR (qRT-PCR) experiment. The histone H2B gene (Eucgr.B03925) was used as the internal control. The forward and reverse primers were predicted using Prime3 locally (https://github.com/primer3-org/primer3.git) and the results with highest score were selected and then synthesized in BGI-Shenzhen. At each timepoint for each biological replicate used in the proteomics and RNA-Seq, we performed three technical replicates in the qRT-PCR experiment, thus, we obtained 9 reactions for every sample of the two eucalyptus species during the callus induction and development. As previously described [8], we normalized the expression of genes in other samples to A1 and used relative normalized expression (RNE) to present the expression of these genes in the two eucalyptus species during the callus induction and development.

## Results

### *Eucalyptus* callus induction

To understand the molecular changes in the eucalyptus species during the somatic embryogenesis, we performed the tissue-culture experiment of stem tissues of two eucalyptus species – *E. camaldulensis* (high embryogenic potential, A1) and *E. grandis* x *urophylla* (low embryogenic potential, B1) (Fig. 1A). After 10 days incubation on CIM, the stem tissues were developed to primary callus (pri-callus, A2 and B2 for *E. camaldulensis* and *E. grandis* x *urophylla*, respectively). Then, mature callus (mat-callus, A3 and B3) was formed after another 11 days incubation on CIM. It is interesting that the induction time on CIM has great impact on the regeneration ability of eucalyptus, which peaked at 21 days of incubation on CIM for both eucalyptus species [8]. We also confirmed that the regeneration rate of *E. camaldulensis* callus (68.89%) was much higher than *E. grandis x urophylla* (3.33%). Then, the mature callus tissues were transferred to SIM for further incubation. The mature callus was developed to the shoot regeneration stage callus (SRS-callus, A4 and B4) with buds after 7 days incubation on SIM and 80% of the mature callus can generate buds after 10 days incubation. However, if the mature callus was incubated on CIM, it was developed to the senescence callus (sen-callus, A5 and B5), which turned brown intensively and lost the regeneration ability.

## *Eucalyptus* gene expression profiles during callus induction and development

Next, we performed the transcriptome sequencing for the stem and callus tissues of the two eucalyptus species. It generated a total of 1,291.85 million raw reads for all samples and 1,003.09 million clean reads (33.44 million reads on average). Genome mapping analysis showed that 69.27% ~ 84.58% of the clean reads can be aligned to the eucalyptus reference genome. Then, we profiled the gene expression in these samples and identified 16,705 and 16,614 genes in *E. camaldulensis* (12,229 to 14,001 genes in A1 to A5) and *E. grandis* x *urophylla* (12,464 to 14,075 genes in B1 to B5), respectively, with average TPM > 5 (Fig. 1B, Supplementary Table S1). The heatmap of correlation between samples (Supplementary Figure S1) showed that SE of *E. grandis* x *urophylla* did not process more slowly than the SE of *E. camaldulensis*. Figure 1B also showed that more than 85% of the identified genes were commonly detected in the two eucalyptus species at the same SE developmental stage. We next performed the differential expression analysis for these samples and identified 5,124, 3,790, 3,111, 4,377, 5,040, 3,740, 4,295, and 2,358 DEGs in A2_vs_A1, A3_vs_A2, A4_vs_A3, A5_vs_A3, B2_vs_B1, B3_vs_B2, B4_vs_B3, and B5_vs_B3, respectively (Fig. 1C, Supplementary Table S2). It is interesting that DEGs varied at different developmental stages during the SE of the two eucalyptus species (Fig. 1D). The KEGG pathway enrichment analysis of the DEGs (Table 1) also showed that different signal transduction pathways were involved in the two eucalyptus species during SE. This indicates that different TFs might be involved in the SE of eucalyptus at different stages. Thus, we searched the DEGs and identified a total of 307 TFs, including 54 WRKY, 51 bHLH, 39 ER (ethylene-responsive), 38 MYB, 9 GATA, 8 NUC (nuclear TF Y subunit), 8 bZIP, 6 GTE, 6 TGA, 5 HS (heat stress), 5 PIF, 4 DIVARICATA, 4 KAN, 4 LHW, 4 NAC, 4 RAX, 4 VRN 4 WER TF genes, with different expression during the SE of the two eucalyptus species (Supplementary Table S3).

**Table 1:** Signal transduction pathways regulated by the DEGs in the somatic embryogenesis of the two eucalyptus species

ID	Pathway	A2 vs A1		A3 vs A2		A4 vs A3		A5 vs A3		B2 vs B1		B3 vs B2		B4 vs B3		B5 vs B3	
		N	FDR	N	FDR	N	FDR	N	FDR	N	FDR	N	FDR	N	FDR	N	FDR
ko04016	MAPK signaling pathway - plant	-	-	-	-	-	-	94	0.0232	-	-	-	-	102	0.00037	-	-
ko04071	Sphingolipid signaling pathway	-	-	-	-	-	-	43	6E-05	46	7.9E-05	-	-	40	0.00118	-	-
ko04152	AMPK signaling pathway	-	-	-	-	-	-	42	5.9E-05	40	0.00696	-	-	-	-	-	-
ko04370	VEGF signaling pathway	-	-	-	-	-	-	-	-	21	0.00513	-	-	-	-	-	-
ko04371	Apelin signaling pathway	-	-	28	0.0401	-	-	-	-	35	0.0106	-	-	-	-	-	-
ko04072	Phospholipase D signaling pathway	32	0.0006	-	-	-	-	26	0.0258	34	4E-05	-	-	-	-	-	-
ko04066	HIF-1 signaling pathway	36	0.00073	-	-	24	0.0266	35	9.3E-05	39	1.9E-05	28	0.00356	-	-	-	-
ko04068	FoxO signaling pathway	44	3.5E-08	31	0.00019	25	0.00662	41	2.9E-08	44	2E-08	27	0.00691	-	-	-	-
ko04075	Plant hormone signal transduction	-	-	127	1.2E-08	125	4.2E-13	137	2.3E-07	147	9.7E-07	125	3.2E-09	154	1.6E-12	84	6.84E-06

## *Eucalyptus* protein changes during callus induction and development

**Fig. 2:**
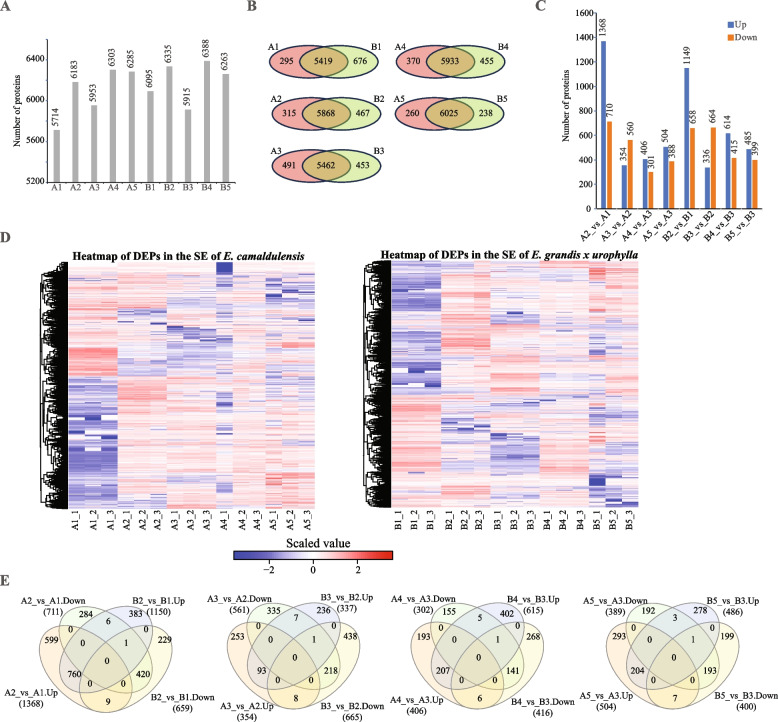
DIA proteomics analysis of the two eucalyptus species during SE. **A** Number of proteins identified in the two eucalyptus species at each stage of the SE process. **B** Venn diagrams of proteins identified in the two eucalyptus species at the same stage of the SE process. **C** Number of DEPs identified in the SE process of the two eucalyptus species. **D** Heat maps of DEPs identified in the two eucalyptus species during SE. **E** Venn diagrams of DEPs identified in the SE process of the two eucalyptus species

Then, we performed DIA proteomics analysis for the same material as transcriptome sequencing. The 33,383 peptides corresponding to 7,238 proteins (5,714 to 6,388 proteins in A1~A5 and B1~B5) were identified in the SE of the two eucalyptus species (Fig. 2A). Heatmap of the protein expression profiles of these samples (Supplementary Figure S2) also confirmed the relationship between samples and no time difference existing in the developmental stages of the two eucalyptus species. We also found that 5,419 to 6,025 proteins were commonly detected in the SE of two eucalyptus species at the same developmental stages (Fig. 2B). Next, we performed differential expression analysis and identified 2,078, 914, 707, 892, 1,807, 1,000, 1,029 and 884 DEPs in A2_vs_A1, A3_vs_A2, A4_vs_A3, A5_vs_A3, B2_vs_B1, B3_vs_B2, B4_vs_B3 and B5_vs_B3, respectively (Fig. 2C, Supplementary Table S4). Heatmaps of DEPs in the SE of *E. camaldulensis* and *E. grandis* x *urophylla* showed that a number of DEPs were specifically expressed in the early SE (Fig. 2D). In addition, venn diagrams showed that at each developmental stage there were specific DEPs in the two eucalyptus species (Fig. 2E). For example, 608, 261, 199 and 300 proteins were specifically upregulated in A2_vs_A1, A3_vs_A2, A4_vs_A3 and A5_vs_A3, respectively. Next, we found 35 TF proteins differentially expressed in the SE of these two eucalypti (Table 2, Supplementary Table S4). It is interesting that differentially expressed TF proteins varied a lot in the SE of the two eucalyptus species from stem to sen-callus. For example, eleven TF proteins were upregulated in A2_vs_A1 but only two of them were upregulated in B2_vs_B1; none of the DEPs encoding TFs in A3_vs_A2 were found in B3_vs_B2; and only one differentially expressed TF protein was shared by A5_vs_A3 and B5_vs_B3. Compared to transcriptome data, results generated by proteomics revealed more difference between the *E. camaldulensis* and *E. grandis* x *urophylla*.

**Table 2:** Differentially expressed TF proteins in the SE of the two eucalyptus species

Protein	A2 vs A1	A3 vs A2	A4 vs A3	A5 vs A3	B2 vs B1	B3 vs B2	B4 vs B3	B5 vs B3	Description
XP_010031175.1	Up	None	None	None	-	Up	None	None	transcription factor BIM1 isoform X1
XP_010036354.1	Up	Down	None	None	None	None	None	None	transcription factor TGA2.3 isoform X1
XP_010044878.1	Up	None	None	Up	Up	None	None	Up	trihelix transcription factor ASR3 isoform X2
XP_010048982.1	Up	-	-	-	None	-	-	-	transcription factor HY5
XP_010052424.1	Up	Down	None	None	None	None	None	None	transcription factor VOZ1 isoform X1
XP_010055248.1	Up	None	None	-	None	None	None	-	bZIP transcription factor 16
XP_010055972.1	Up	None	None	None	None	None	None	None	transcription factor TGA2.3
XP_010060108.1	Up	Down	None	None	None	None	None	None	GATA transcription factor 18 isoform X1
XP_018716037.1	Up	Down	None	Up	Up	None	None	None	probable transcription factor At3g04930, partial
XP_018730947.1	Up	None	None	None	-	None	None	None	transcription factor SOX-30
XP_018731692.1	Up	None	None	None	None	None	None	Up	probable WRKY transcription factor 40
XP_010024394.1	None	None	None	None	Up	None	None	None	nuclear transcription factor Y subunit C-9-like
XP_010050871.1	None	None	None	None	None	Down	None	None	transcription factor-like protein DPB isoform X1
XP_010054774.1	None	Down	Up	None	None	None	None	None	transcription factor MYB51
XP_010055446.1	None	Down	None	None	-	-	None	Up	ethylene-responsive transcription factor 2
XP_010057098.1	None	Down	None	None	None	None	None	None	trihelix transcription factor ASIL2-like isoform X1
XP_010059753.1	None	None	-	None	Up	Down	-	None	transcription factor MYB44
XP_010062128.1	None	-	-	-	None	Down	-	None	transcription factor VIP1
XP_010063839.1	None	None	None	Up	None	None	None	None	ethylene-responsive transcription factor ABR1
XP_010067716.1	None	Down	None	None	None	None	None	None	transcription factor TGA2
XP_010027780.1	Down	-	-	-	Down	-	-	-	nuclear transcription factor Y subunit B-3-like
XP_010032813.1	Down	None	None	None	None	None	None	None	transcription factor Pur-alpha 1 isoform X1
XP_010061728.1	Down	None	None	Up	Down	None	None	None	ethylene-responsive transcription factor RAP2-1
XP_010062114.1	Down	-	-	-	-	-	-	-	trihelix transcription factor GT-2 isoform X1
XP_010066464.1	Down	None	None	None	-	None	None	None	ethylene-responsive transcription factor ERF113
XP_010023972.1	-	Down	-	None	-	None	-	Down	ethylene-responsive transcription factor ERF096
XP_010024807.1	-	-	-	-	Up	None	-	None	probable WRKY transcription factor 23
XP_010025536.1	-	-	None	-	-	-	Up	None	ethylene-responsive transcription factor ERF110
XP_010036765.1	-	-	-	-	Down	None	None	None	transcription factor bHLH62
XP_010048564.1	-	-	-	-	Down	-	-	-	transcription factor bHLH35 isoform X2
XP_010049827.1	-	Up	None	None	-	None	-	None	probable WRKY transcription factor 17
XP_010060240.1	-	None	-	Up	None	None	None	None	probable WRKY transcription factor 20
XP_010060309.2	-	None	None	None	-	Down	None	None	WRKY transcription factor 42
XP_010060857.1	-	None	None	None	-	Down	None	Up	nuclear transcription factor Y subunit C-2
XP_010063696.1	-	-	-	Up	-	-	-	-	myb family transcription factor PHL6

### Combination analysis of the transcriptome and proteome data

**Fig. 3:**
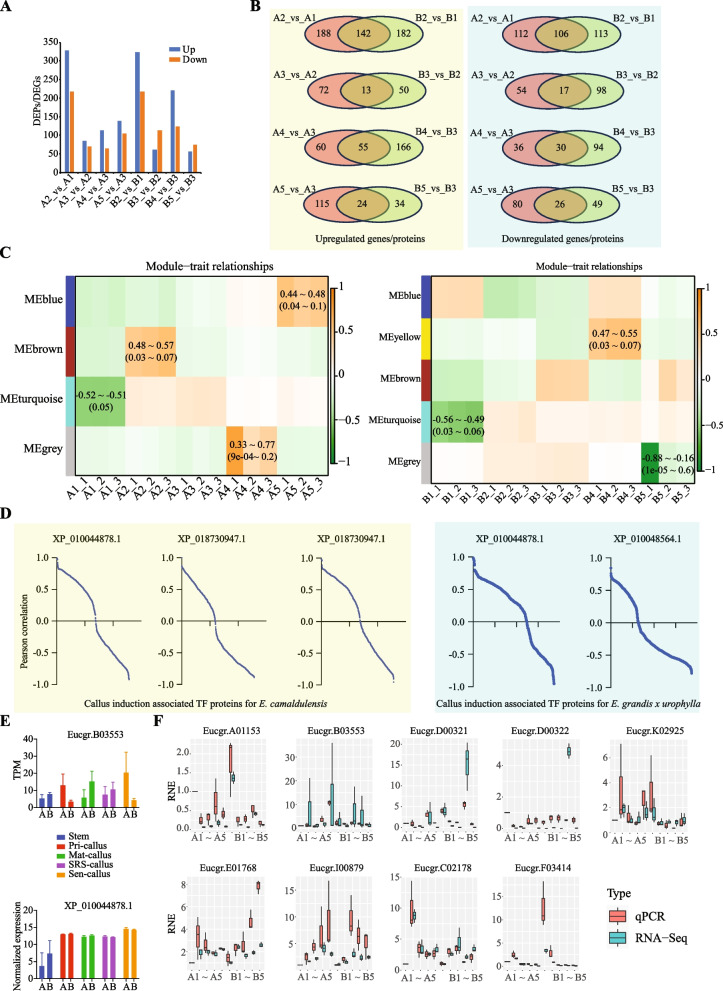
Combination analysis of the transcriptome and proteome data. **A** Commonly DEPs/DEGs in the SE process of the two eucalyptus species identified by DIA proteomics and transcriptome sequencing technologies. **B** Comparison of commonly DEPs/DEGs identified in the SE of *E. camaldulensis* and *E. grandis* x *urophylla* at each stage. **C** WGCNA of the DEPs/DEGs identified in the two eucalyptus species. Left: *E. camaldulensis*; right: *E. grandis* x *urophylla*. **D** Correlation analysis of DEPs/DEGs and three TFs (XP_010044878.1, XP_018730947.1 and XP_010062114.1) in the callus induction of *E. camaldulensis* and *E. grandis* x *urophylla*. **E** Gene expression (upper panel) and protein expression (lower panel) levels of Eucgr.B03553 and its protein XP_010044878.1 in the two eucalyptus species during SE. **F** qRT-PCR validation results for 7 candidate genes

Then, we tried to incorporate the transcriptome and proteome results and find the key molecules involved in the SE induction and development of the two eucalyptus species. As shown in Fig. 3A, there were 1,353 (131 to 546) DEGs/DEPs identified by both transcriptome sequencing and the DIA proteomics (Supplementary Table S5). It is interesting that 142 genes/proteins commonly upregulated during the callus induction of the two eucalyptus species, however, only 13 and 55 genes/proteins were commonly upregulated in the callus maturation and development (Fig. 3B). It is notable that the most significant pathways were the metabolism associated pathways involved by either the upregulated or the downregulated genes/proteins during the callus induction and development of the two eucalyptus species (Supplementary Table S6). In addition, several signaling pathways were also involved by the DEGs/DEPs. For example, 4 downregulated genes/proteins were enriched in the MAPK signaling pathway (plant) in the callus maturation of the two eucalyptus species; FoxO signaling pathway was specifically deregulated during the callus development of *E. camaldulensis*.

Next, we identified eight differentially expressed TF genes/proteins commonly detected by transcriptome sequencing and the DIA proteomics (Supplementary Table S5). Among them, XP_010044878.1~Eucgr.B03553 (trihelix transcription factor ASR3 isoform X2) was commonly upregulated in the callus induction of the two eucalyptus species, another two TFs (SOX30 and WRKY) were specifically upregulated in the callus induction of *E. camaldulensis*, three TFs (bHLH62, bHLH35 isoform X2 and RAP2-1) were specifically downregulated in the callus induction of *E. grandis* x *urophylla*, and XP_010050871.1~Eucgr.A01674 (transcription factor-like protein DPB isoform X1) was specifically downregulated during the callus maturation of *E. grandis* x *urophylla*. The expression patterns of these TFs identified by the transcriptome sequencing and DIA proteomics strongly support that they might be associated with the SE capacity of eucalyptus. While more experiments are required to explore their functions and regulatory network during the callus induction and development in eucalyptus.

### WGCNA

To identify co-expressed genes/proteins within the callus induction and development of the two eucalyptus species, we performed WGCNA and identified 4 and 5 modules for *E. camaldulensis* and *E. grandis* x *urophylla*, respectively (Fig. 3C). Further, modules of turquoise, brown, grey and blue were significantly associated with the stem, pri-callus, SRS-callus and sen-callus, respectively, of *E. camaldulensis*. Notably, two upregulated (XP_010044878.1 and XP_018730947.1) and one downregulated (XP_010062114.1) TF was identified in the turquoise module of genes/proteins that might be closely associated with the callus induction of *E. camaldulensis*. Further, we found that the expression of 125, 124 and 138 DEGs/DEPs was highly correlated (Pearson correlation > 0.8 or < -0.8) with the expression of TFs XP_010044878.1, XP_018730947.1 and XP_010062114.1, respectively (Fig. 3D). No TF proteins were found in the brown, grey and blue modules for *E. camaldulensis*. Next, we analyzed the TFs and their co-expressed genes/proteins for *E. grandis* x *urophylla* during the callus induction and development. The turquoise, yellow and grey modules of DEGs/DEPs were found to be significantly associated with the stem, SRS-callus and sen-callus, respectively, of *E. grandis* x *urophylla* (Fig. 3C). Interestingly, only two TF proteinss (XP_010044878.1 and XP_010048564.1) were found to be associated with the callus induction of *E. grandis* x *urophylla*. Their expression was found to significantly correlated with 26 DEGs/DEPs (25 and 1 for XP_010044878.1 and XP_010048564.1, respectively) (Fig. 3D). It is notable that XP_010044878.1 (trihelix transcription factor ASR3 isoform X2) was identified in the callus induction for both eucalyptus species. The protein expression of XP_010044878.1 had no significant difference between the two eucalyptus species during the SE, however, its gene Eucgr.B03553 was differentially expressed between the eucalyptus species during the SE, especially at the callus induction (Fig. 3E). Eucgr.B03553 was upregulated in *E. camaldulensis* but downregulated in *E. grandis* x *urophylla* during the callus induction. In addition, the XP_010044878.1 correlated genes identified by WGCNA for the two eucalyptus species only had one in common - Eucgr.E03807 (external alternative NAD(P)H-ubiquinone oxidoreductase B2, mitochondrial), which was downregulated in *E. grandis* x *urophylla* (Supplementary Table S5). These results indicated that the XP_010044878.1 may regulate different genes during the callus induction and development of the two eucalyptus species, which might be associated with the embryogenic potential. While more experiments are required to confirm the key functions of XP_010044878.1 in the SE of eucalyptus and its relationship with the embryogenic potential of eucalyptus.

### Genes/proteins in plant hormone signal transduction and MAPK signaling pathways

**Fig. 4:**
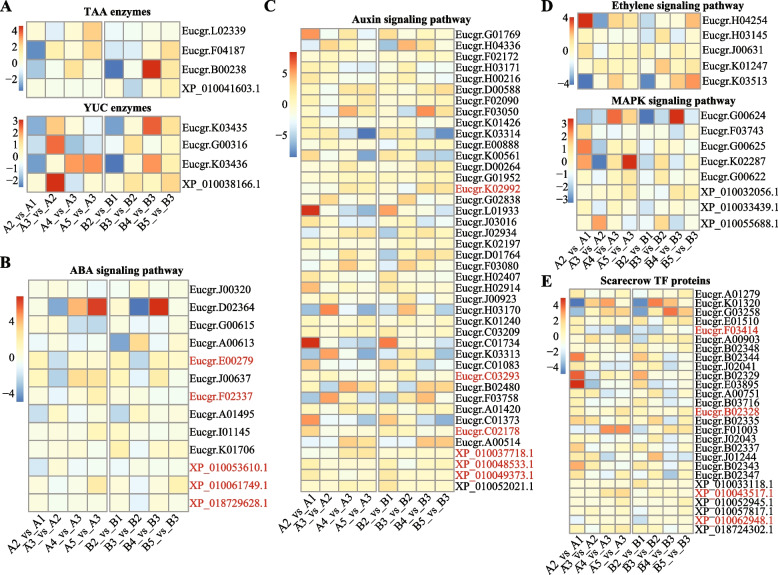
DEGs/DEPs involved in the plant hormone transduction and MAPK signaling pathways. **A** Differentially expressed tryptophan aminotransferase and YUCCA enzyme associated genes and proteins in the two eucalyptus species during SE. **B** DEGs/DEPs from ABA signaling pathway in the SE of the two eucalyptus species. **C** DEGs/DEPs from the auxin signaling pathway in the SE of the two eucalyptus species. **D** DEGs/DEPs from the ethylene and MAPK signaling pathways in the SE of the two eucalyptus species. **E** Differentially expressed scarecrow TF genes and proteins (from the plant hormone transduction pathway) in the SE of the two eucalyptus species. Genes and proteins in red represent that the expression change of the gene/protein was confirmed by both transcriptome sequencing and proteomics

We next further identified 3, 3, 281 and 378 DEGs/DEPs associated with tryptophan aminotransferase (TAA) and YUCCA (YUC) enzymes (like indole-3-pyruvate monooxygenase [EC:1.14.13.168]) [27], plant hormone transduction pathway and MAPK signaling pathway in the SE of the two eucalyptus species (Supplementary Table S7). The TAA and YUC enzymes are key molecules in the auxin biosynthesis, we found that Eucgr.K03435 (YUCCA10) showed downregulation in both eucalyptus species (Fig. 4A), and that Eucgr.F04187 (tryptophan aminotransferase-related protein 3), Eucgr.G00316 (YUCCA10) showed specific downregulation in *E. camaldulensis* during the process of stem induced to pri-callus (Fig. 4A).

Then, we looked at the genes/proteins involved in the plant hormone transduction pathway. There were 13 DEGs/DEPs involved in the abscisic acid (ABA) signaling pathway (Fig. 4B, Supplementary Table S7), including 6 ABA receptors (Eucgr.A00613, Eucgr.A01495, Eucgr.D02364, Eucgr.E00279, XP_010053610.1, XP_018729628.1) and 7 ABA-insensitive 5 like proteins (Eucgr.J00320, Eucgr.J00637, Eucgr.K01706, Eucgr.F02337, Eucgr.G00615, Eucgr.I01145, XP_010061749.1). Notably, the expression changes of two ABA receptors (XP_010053610.1, XP_018729628.1~Eucgr.E00279) and one ABA-insensitive 5 like protein 5 (XP_010061749.1~Eucgr.F02337) were confirmed by both transcriptome sequencing and proteomics (Fig. 4B). XP_010053610.1~Eucgr.D02201 was specifically downregulated in the callus induction of *E. camaldulensis*, XP_018729628.1~Eucgr.E00279 was specifically downregulated in the callus induction of *E. grandis* x *urophylla*, and XP_010061749.1~Eucgr.F02337 was specifically upregulated in the senescence process of *E. camaldulensis*.

We next identified 42 DEGs/DEPs involved in the auxin signaling pathway (Fig. 4C, Supplementary Table S7), including 15 ARFs, 5 auxin transporter-like proteins, 5 auxin-induced proteins, 16 auxin-responsive proteins and one auxin-singling protein F-BOX 2. While only the changes of XP_010049373.1~Eucgr.C03293 (auxin response factor 19 isoform X2), XP_010048533.1~Eucgr.C02178 (auxin response factor 19 isoform X1) and XP_010037718.1~Eucgr.K02992 (auxin transporter-like protein 4) were identified by both transcriptome sequencing and proteomics (Supplementary Table S5). Notably, XP_010048533.1~Eucgr.C02178 was upregulated in the callus induction from stem to pri-callus in both eucalyptus species, XP_010049373.1~Eucgr.C03293 was specifically downregulated in the callus development of *E. camaldulensis*, and XP_010037718.1~Eucgr.K02992 was downregulated in the callus induction process but upregulated in the callus development of *E. camaldulensis*.

Interestingly, the six ABA receptor genes/proteins were also involved in the MAPK signaling pathway (Supplementary Table S7). Further, we identified 5 ethylene signaling associated genes and 8 MAPKs (MMK2, MAPK3, MAPK7, MKK9, MAP3K1) involved in the MAPK pathway (Fig. 4D, Supplementary Table S7), however, their expression patterns were detected only by the transcriptome sequencing. It is notable that 50 WRKY TF DEGs/DEPs were involved in the MAPK pathway (Supplementary Table S7), while only expression change of XP_018731692.1~Eucgr.F03955 (WRKY40) was confirmed by both technologies and specific to the callus induction of *E. camaldulensis*.

In addition, we identified 27 DEGs/DEPs related to the SCARECROW TF proteins in the SE of the two eucalyptus species (Fig. 4E, Supplementary Table S7). However, only the downregulation of XP_010062948.1~Eucgr.F03414 (scarecrow-like protein 21) that was confirmed by both transcriptome sequencing and proteomics was found specifically in the callus induction of *E. grandis* x *urophylla*. Another scarecrow-like protein 30 (XP_010043517.1~Eucgr.B02328) was found with upregulation in the senescence of *E. camaldulensis*.

When we compared the key molecules mentioned above with the possible genes/proteins regulated by XP_010044878.1, Eucgr.C02208 (bHLH35) from both plant hormone transduction and MAPK signalling pathways and XP_010055417.1 (dihydrofolate reductase) from the MAPK signalling pathway were identified (Supplementary Table S7). The bHLH35 TF gene was commonly downregulated in the early callus induction and callus development processes of both eucalyptus species. XP_010055417.1 was commonly upregulated in the late callus induction process of both eucalyptus species. These evidence supports that they might be downstream targets of XP_010044878.1 and function in the SE of eucalyptus.

### qRT-PCR validation

We performed qRT-PCR to validate the expression patterns of DEGs in the SE of eucalyptus, including Eucgr.C02178 (*ARF19*), Eucgr.F03414 (scarecrow-like protein 21), Eucgr.B03553 and genes (Eucgr.A01153, Eucgr.D00321, Eucgr.D00322, Eucgr.E01768, Eucgr.I00879 and Eucgr.K02925) correlated with Eucgr.B03553 during the callus induction and development of the two eucalyptus species. To compare their expression patterns detected by qRT-PCR and RNA-Seq, we normalized their expression levels to A1 and showed their relative normalized expression (RNE) in Fig. 3F. The expression patterns of 8 genes (88.9%), including Eucgr.B03553, showed high agreement by both RNA-Seq and qRT-PCR, indicating that the genes identified in this study might be associated with the callus induction and development of the two eucalyptus species. Taking all together, Eucgr.B03553 might be a key regulator that is associated with high embryogenesis potential of eucalyptus species and can play a fundamental role during the callus induction and development of *E. camaldulensis*.

## Discussion

Three major stages have been reported in the eucalyptus SE from tissue culture, including co-cultivation, callus induction and shoot regeneration [28]. In this study, we not only set up the induction and callus development (similar to the shoot regeneration) for the two eucalyptus species, but also used the sen-callus as negative control for the callus development (Fig. 1A). This enables the discovery of key regulators involved in the callus development. From the transcriptomics and proteomics results, we identified vary genes (Fig. 1C, Supplementary Table S2) and proteins (Fig. 2C, Supplementary Table S4) that might be associated with the callus induction and development. Among them, 142,13 and 55 genes/proteins were commonly upregulated during the callus induction, maturation and development stages, respectively, of the two eucalyptus species (Fig. 3B). Previously reported SE associated molecules were also found in this study, such as *SERK2*, *ARF19* and *WUS* [29, 30]. However, they were detected with different expression patterns in the two eucalyptus species. For example, WUS (Eucgr.A02149/NP_001289667.1) and ARF19 (Eucgr.C02178/XP_010048533.1) were upregulated during the callus induction process in the two eucalyptus species from both transcriptome and proteome results, however, SERK1 (Eucgr.H03383/ XP_010024200.1) was upregulated only in the callus induction of *E. camaldulensis* (Supplementary Table S2, Supplementary Table S4). Our results confirmed that these genes are key regulators for the SE in eucalyptus and that SERK1 might be associated with high SE potential of *E. camaldulensis*.

We also identified the trihelix transcription factor ASR3 (XP_010044878.1, trihelix transcriptional factor ARABIDOPSIS SH4-RELATED 3) as a key regulator during the SE of the two eucalyptus species (Fig. 3). There are five sub-families of trihelix TF including GT-1, GT-2, GT-γ, SH4 and SIP1 [31], however, very few studies have been demonstrated to investigate their association with callus and embryo development in plant. The ASR3 TF identified in this study is from the SH4 family and has been reported to play an important role during the activation of cell differentiation in rice [32]. Previous study has shown that ASR3, regulated by MAMP-activated MPK4, functions as a transcriptional repressor to fine-tune plant immune gene expression in Arabidopsis [33]. In addition, ASR1 together with its interacting TF 1 (AITF1) can directly activate the SAA1 expression through binding to the GT-boxes in SAA1 promoter and further modulate the plant disease resistance and autoimmunity [34]. In our study, ASR3 protein was upregulated in the callus induction of the two eucalyptus species (Fig. 3E), indicating its important functions in this process. Further, we found that ASR3 might regulate 25 DEGs/DEPs correlated with ASR3 during the callus induction and development in the two eucalyptus species (Fig. 3D). Interestingly, there was another trihelix TF identified with downregulation specifically to *E. camaldulensis* during the callus induction (Supplementary Table S5) and it has been reported to be involved in cold, drought, and salt stress responses in plants [35, 36]. Their deregulation and high correlation strongly support that they might be functional during the SE of two eucalyptus species.

In addition, SOX30 and WRKY40 TFs were specifically upregulated in the callus induction of *E. camaldulensis*, three TFs (bHLH62, bHLH35 isoform X2 and RAP2-1) were specifically downregulated in the callus induction of *E. grandis x urophylla* (Supplementary Table S5). It is interesting that SOX30, a member of the SOX (SRY-related HMG-box) family of transcription factors, has been reported to be involved in the regulation of embryonic development and in the determination of the cell fate in mouse [37]. We were not surprised that the super WRKY TF family was involved in the callus induction of Eucalyptus and related to the SE potential, because the members of WRKY TF family can often act as repressors as well as activators and play important roles in many biological processes in plants [38]. While WRKY11 was found with upregulation in heat-priming maritime pine (*Pinus pinaster*) megagametophytes during SE [39]. In rubber tree, WRKY40 was also identified as a key regulator for the SE [13]. The high expression of SOX30 and WRKY40 TFs might be related to the high SE potential of *E. camaldulensis*. On the other hand, the downregulation of bHLH and RAP2-1 TFs might also be responsible for the low SE potential of *E. grandis x urophylla*. The bHLH TFs have been reported to be expressed and play important roles in the embryogenic callus of many plants, such as papaya [40], eucalyptus [8], and cotton [41]. Like bHLH TFs, RAP2 has also been identified as a key regulator during the SE of plants. RAP2.6L was identified as a key factor for shoot regeneration in Arabidopsis. As one of the ethylene signal transduction related genes, RAP2-3 was identified with upregulation by Wang *et al.* in the cotyledonary embryo during SE of rubber [13]. These results indicated that the TFs and other molecules identified in this study might be associated with the high SE potential of eucalyptus, however, further experiments are required to explore their molecular functions in the SE.

## Conclusions

We used transcriptomics and proteomics techniques to identify the transcription factors and their possible downstream genes during the somatic embryogenesis of two eucalyptus species. Like other studies, ARF19 and SERK2 were found to be significant regulators of the eucalyptus SE, further, we identified the trihelix transcription factor ASR3 isoform X2 as a potential key regulator of the callus induction process of eucalyptus. DEGs/DEPs involved in the plant hormone transduction (ABA and auxin signaling) and MAPK signaling pathways showed different expression patterns in the SE of the two eucalyptus species. Compared with our previous studies, in the present study we found that the scarecrow-like TF family might be key regulators of the eucalyptus SE, like XP_010062948.1~Eucgr.F03414 (scarecrow-like protein 21). Further, we first reported that SOX-30 and WRKY40 were associated with the high embryogenesis potential of *E. camaldulensis*. Further functional experiments are required to validate their functions during somatic embryogenesis in eucalyptus and other plants. The methods would help researchers to study transcription factors using multi-omics data. Our findings will bring new insights into the molecular mechanisms and gene regulations for plant SE studies, especially eucalyptus. Further, the output will benefit the eucalyptus breeding program.

### Supplementary Information

Supplementary Material 1.

Supplementary Material 2.

Supplementary Material 3.

Supplementary Material 4.

Supplementary Material 5.

Supplementary Material 6.

Supplementary Material 7.

Supplementary Material 8.

## Data Availability

The original files of DDA and DIA MS data can be accessed from the iProx website under the accession numbers IPX0004336001 and IPX0004336002. The raw data of transcriptome sequencing can be accessed from the NCBI Sequence Read Archive (SRA) platform (http://trace.ncbi.nlm.nih.gov/Traces/sra/) under the accession number PRJNA761197 and PRJNA634476.

## Abbreviations

SE: somatic embryogenesis
TF: transcription factor
DEP: differentially expressed proteins
DEG: differentially expressed genes
CIM: callus induction MS medium
SIM: shooting-inducing medium
TPM: transcripts per million reads mapped
log2FC: log2 fold change
DDA: data dependent acquisition
DIA: data independent acquisition
GO: gene ontology
KEGG: Kyoto Encyclopedia of Genes and Genomes
WGCNA: Weighted correlation network analysis
RNE: relative normalized expression;
pri-callus: primay callus
mat-callus: mature callus
SRS-callus: shoot regeneration stage callus
sen-callus: senescence callus
NUC: nuclear TF Y subunit
ER: ethylene-responsive
HS: heat stress
