# Supplementary material for: Integration analysis of transcriptome and proteome profiles brings new insights of somatic embryogenesis of two eucalyptus species
Source: BMC Plant Biol. 2024 Jun 15;24:561. doi: 10.1186/s12870-024-05271-6 (PMC11179386; doi:10.1186/s12870-024-05271-6)
Supplement: Supplementary file 2 — Supplementary Material 2. [file 12870_2024_5271_MOESM2_ESM.pdf]

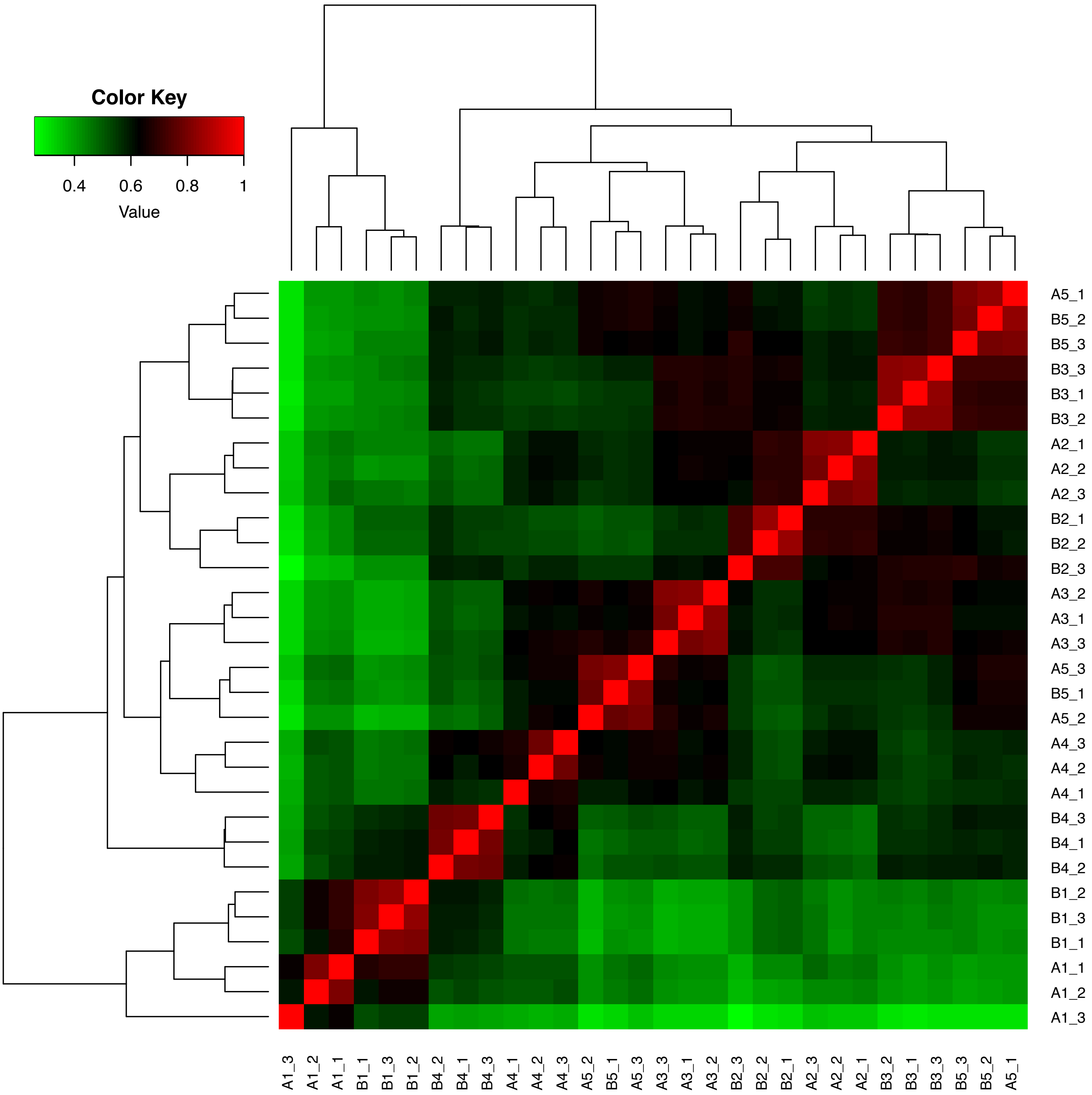

Supplementary Figure S2. Heat map of correlation analysis of the samples based on the protein expression profiles.
